# Ant Homing Ability Is Not Diminished When Traveling Backwards

**DOI:** 10.3389/fnbeh.2016.00069

**Published:** 2016-04-13

**Authors:** Paul B. Ardin, Michael Mangan, Barbara Webb

**Affiliations:** Insect Robotics Lab, School of Informatics, University of EdinburghEdinburgh, UK

**Keywords:** ants, navigation, visual homing, insect, retinotopic, view matching

## Abstract

Ants are known to be capable of homing to their nest after displacement to a novel location. This is widely assumed to involve some form of retinotopic matching between their current view and previously experienced views. One simple algorithm proposed to explain this behavior is continuous retinotopic alignment, in which the ant constantly adjusts its heading by rotating to minimize the pixel-wise difference of its current view from all views stored while facing the nest. However, ants with large prey items will often drag them home while facing backwards. We tested whether displaced ants (*Myrmecia croslandi*) dragging prey could still home despite experiencing an inverted view of their surroundings under these conditions. Ants moving backwards with food took similarly direct paths to the nest as ants moving forward without food, demonstrating that continuous retinotopic alignment is not a critical component of homing. It is possible that ants use initial or intermittent retinotopic alignment, coupled with some other direction stabilizing cue that they can utilize when moving backward. However, though most ants dragging prey would occasionally look toward the nest, we observed that their heading direction was not noticeably improved afterwards. We assume ants must use comparison of current and stored images for corrections of their path, but suggest they are either able to chose the appropriate visual memory for comparison using an additional mechanism; or can make such comparisons without retinotopic alignment.

## 1. Introduction

Non-pheromone laying ants are expert navigators, with individuals capable of foraging for up to 1 km (Huber and Knaden, 2015) before precisely relocating their hidden nest entrance. Normally this behavior is supported by path integration (Müller and Wehner, 1988), but visual cues alone are sufficient to guide individuals home along familiar routes (Wehner et al., 1996; Kohler and Wehner, 2005; Mangan and Webb, 2012). Ants are also capable of using visual information to return from previously unvisited locations following a displacement (Wehner and Räber, 1979; Zeil, 2012). This is commonly termed “visual homing,” and can be explained if there is sufficient overlap of the current scene with stored memories around the target location such that comparison between them allows an appropriate direction of movement to be obtained.

Hypothesized strategies of visual homing in insects generally adhere to a bottom-up methodology: seeking the simplest mechanism that can account for the behavior, with complexity only added when required to explain new behavioral data (Wystrach and Graham, 2012). A widespread simplifying assumption is that ants' visual memory is a retinotopic snapshot (Collett and Cartwright, 1983; Wehner et al., 1996), rather than a reconstruction of the 3D arrangement of surrounding landmarks see also discussion in Collett et al. (2013). This memory could be a single environmental feature which has strong visual qualities and is acting as a beacon, which the ant aims to keep in the center of the field of view, or at some fixed retinal displacement, for some part of its journey (Collett, 1996). Or it could be the entire panorama (Zeil et al., 2003), or skyline (Graham and Cheng, 2009), or some parametric representation of the image projected on the retina such as the average landmark vector (Möller et al., 2001) or visual center of mass (Hafner, 2001). The assumption that the retinopic projection is important is supported by evidence that changing the distance and size of landmarks in such a way as to present the same view from a given location results in ants treating it as the same location (Wehner and Räber, 1979; Åkesson and Wehner, 2002; Narendra et al., 2007). In addition, evidence that ants do not appear to be able to transfer landmark information acquired in one eye to drive successful guidance when viewing the world with the other eye (Wehner and Müller, 1985), has been taken to support the view that this memory is “fixed relative to retinal co-ordinates” (Wehner et al., 1996) (i.e., cannot be mentally rotated, and is not stored in some rotation invariant form). Thus physical alignment of the animal to the same viewing direction as when the memory was stored must play a role in the homing process (Wehner et al., 1996).

Following such alignment, there are multiple ways the ant could use its visual memory to obtain a heading direction, either through a calculation based on the difference between memory and the current view (Cartwright and Collett, 1982; Möller et al., 2001; Vardy and Moller, 2005; Möller and Vardy, 2006), simple “move and compare” gradient descent on the translational image difference function (tIDF) (Zeil et al., 2003; Stürzl et al., 2008; Mangan, 2011; Stürzl et al., 2015), or through recovery of a “local vector”(Collett, 2010) or a motor action (Lent et al., 2009) associated to the view. The alignment itself could be based on external cues, such as the celestial compass, allowing the animal either to rotate to match a particular stored view, or to chose from multiple stored views the one which is best aligned (Collett and Cartwright, 1983; Mangan and Webb, 2009; Möller, 2012), before attempting to recover the heading. However, a parsimonious alternative is that alignment itself can be driven by view comparison (Zeil et al., 2003). Using a simple pixel-wise image difference function (IDF), as the ant rotates it will experience a minimum in the IDF when its current view is aligned to a visual memory. Moreover, if the memory was stored while the ant was facing the desired heading direction (e.g., facing the nest), then turning to face the minimum in the rotational IDF (rIDF) means the ant has recovered the required heading direction; that is, retinotopic alignment is not just necessary, but sufficient, for visual homing. The observation of “scanning” behaviors in homing ants (rotating on the spot before selecting a direction of travel) (Wystrach et al., 2014; Zeil et al., 2014) seems consistent with this explanation.

An important generalization of this idea is that the ant may be able to use the rIDF to recover the current best heading direction relative to a large set of memories, without needing to choose a specific memory for the comparison. For example, the smallest pixel-wise difference (or greatest “familiarity”) between all memories and the view experienced during a physical rotation will be found for the spatially closest memory, when view and memory are aligned. The plausibility of this method is supported by neural algorithms for efficient simultaneous storage and comparison of multiple views collected along routes toward the nest (Baddeley et al., 2012; Ardin et al., 2016). Making the additional assumption that this method for recovering the heading is applied continuously by the ant, the retinotopic alignment algorithm can generate long-range route following behaviors in simulated ant environments (Baddeley et al., 2012; Ardin et al., 2016). In addition by training the same algorithm with multiple homeward facing memories, as might have been obtained on learning walks (Nicholson et al., 1999; Graham et al., 2010; Müller and Wehner, 2010), visual homing from novel locations can emerge (Wystrach et al., 2013; Dewar et al., 2014; Zeil et al., 2014; Stürzl et al., 2015). Most recently, Kodzhabashev and Mangan (2015) simplified this approach further by demonstrating that route following is possible using a continuous oscillatory algorithm driven by the instantaneous view familiarity, removing the need for scanning at every step. Some authors, including ourselves, have thus suggested that the ant could have a single mechanism for route following and homing, involving continuous realignment to obtain the best retinotopic match to memories facing the nest (Wystrach et al., 2013; Dewar et al., 2014; Ardin et al., 2016).

However, it should be noted that in some experiments, ants displaced from a familiar route to a nearby novel location do not move in the direction of the rIDF minimum but prefer the heading predicted by the lowest points of the skyline (Wystrach et al., 2012). Perhaps more crucially, ants often do not face the direction they are traveling, for example while undertaking group transport (Czaczkes and Ratnieks, 2013), when being carried by another ant (Fourcassie et al., 2000) and when they need individually to move a cumbersome food item.

In the current study we provide ants with large prey item which they can only deliver to the nest by dragging backwards, and assess their ability to home after displacement while experiencing an inverted viewpoint relative to the direction of travel. The aggressive predatory ant, *Myrmecia croslandi*, is ideal for studying navigation under challenging visual conditions as it will readily attack large prey and inhabits an environment where the both tIDF and rIDF have been shown to be consistent with the paths taken by displaced ants (Narendra et al., 2013; Stürzl et al., 2015). We find that the facing direction of ants—toward or away from the nest—has little influence on their ability to move directly home, which casts some doubt on the hypothesis that continuous retinotopic matching is a sufficient mechanism to explain homing.

## 2. Materials and methods

**Figure 1:**
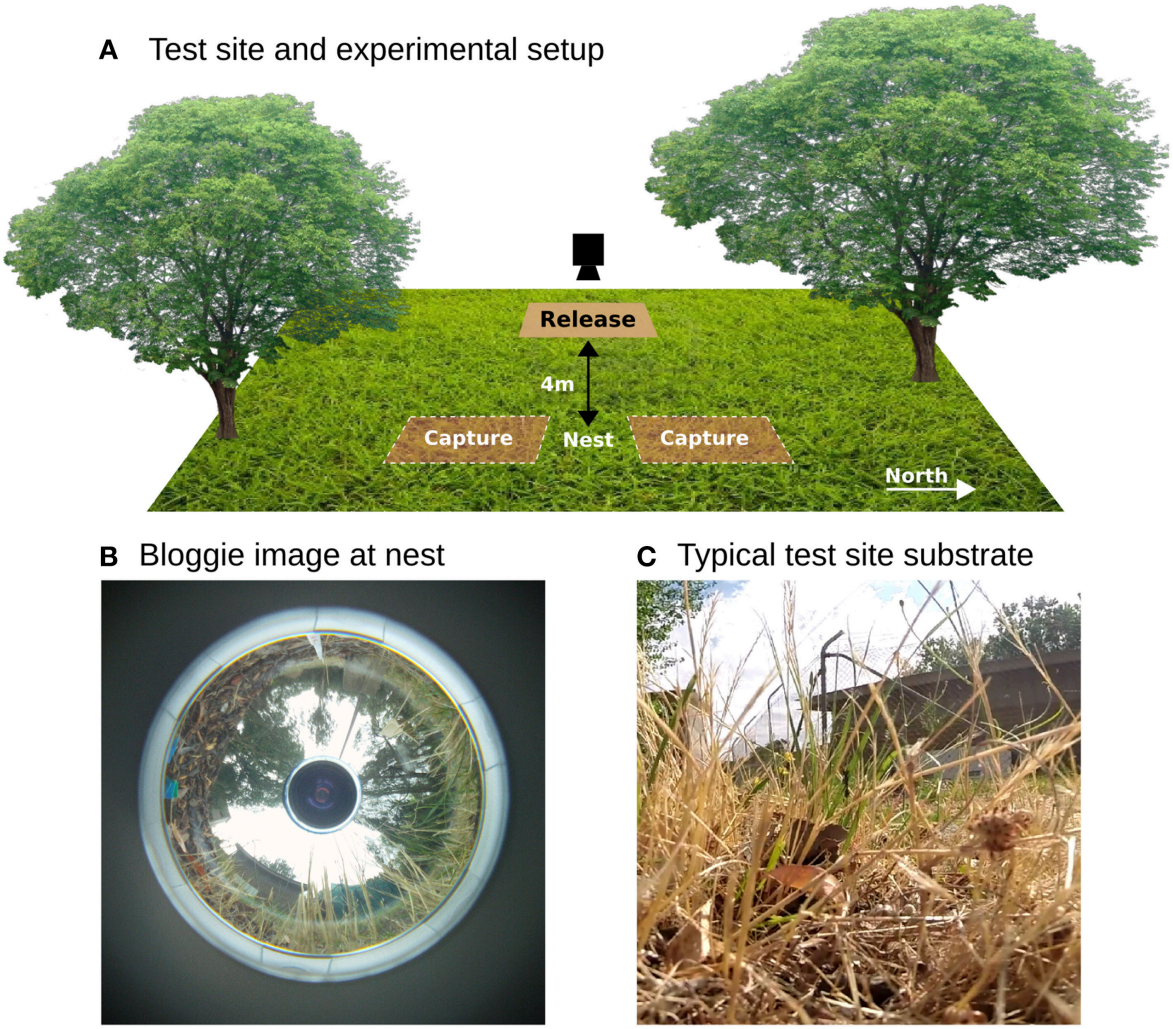
**Overview. (A)** Ants were captured approximately 1–1.5 m from the nest on foraging runs towards the north and south. They were then released on a platform 4m West and the entire homeward track filmed by repositioning a camera on a tripod. **(B)** A panoramic picture taken at the nest using Sony Bloggie camera shows there is a distinctive panorama dominated by the surrounding trees. **(C)** Image from within the grassy substrate highlighting the complex 3D environment through which the ants must navigate by walking or jumping.

Experiments were conducted at the campus field station at the Australian National University (3516′49.8′′ S 14906′43.9′′E). A single nest of *M. croslandi* (colloquially named “Jumping Jacks” due to the frequent jumps they perform even when carrying large food items) was used for all the experiments. The foraging patterns of ants from this nest are characterized in Jayatilaka et al. (2014). Ants collect nectar from two eucalyptus trees located C.10 m South (200 degrees from north) of the nest entrance but the visual panorama is also dominated by another tree C.8 m North (320 degrees) (Figures 1A,B). During the morning and afternoon ants seek prey in the area a few meters around the nest which they subdue with a sting before transporting it back to the nest across rough grass and leaf litter up to 20 cm in depth (Figure 1C).

Outbound foragers were collected at approximately 1–1.5 m from the nest; 12 were traveling South and 8 North. Captured ants were kept in the dark for 15–30 min during which time they were fed a 10% sugar solution. Immediately prior to release, ants were offered a large live prey item (a cricket approximately 1 cm length). Individual ants were then released from a clear test tube on a level platform (50 cm × 85 cm) positioned 4 m West (275 degrees from the nest). This is distant from the familiar route to the tree (Figure 1A) but we cannot be certain that ants have not previously foraged in this direction. A total of 20 ants were tested, of which 8 refused the prey item and ran home unencumbered. The remaining 12 stung the prey before dragging it back to the nest.

The ant paths were recorded from above using either a Sony FDR-AX100 or Panasonic DMC-FZ200 camera mounted on a tripod, which was repositioned repeatedly to capture the path from the point the animal left the test tube all the way back to the nest, with some small gaps during camera repositioning. The position of the ant and its head-tail orientation were determined in every 25th frame (i.e., at 1 s intervals) using custom software (Matlab) with control points marked when the camera was moved. The sections of track recorded in each camera position were then aligned by matching control points and rotating the track around a fixed point (the nest). The difference between successive positions *x*_*i*_ is used to determine the direction of movement of the ant in each frame, and tortuosity of the path calculated as:

(1) $$\begin{array}{l} \frac{\sum \left(x_{i+1}-x_{i}\right)}{x_{n}-x_{1}} \end{array}$$

i.e., the sum of euclidean distances between successive positions on the track divided by the euclidean distance between the start and end of the track.

An ant was considered as facing forwards when the head-tail orientation was within an arc ±90 degrees centered on the bearing of the nest. Using this definition, the length of segments of track facing continuously away from or toward the nest could be calculated. We note that these ants have been observed in previous studies (Zeil et al., 2014) to show substantial independent movement of the head relative to the body (which we could not resolve for the resolution in our video) which could introduce up to 30 degrees difference between the head-tail orientation and the actual gaze direction of the ant. Nevertheless, we can safely assume an ant with a backward orientation (more than ±90 degrees from the nest bearing) could not be gazing directly at the nest.

For one of the analyses below, we assume that ants could be gazing at the nest (i.e., obtaining retinal alignment with a image stored when facing the nest from the same direction) each time their head-tail orientation falls below ±5 degrees of the nest. We then average their direction of movement relative to the nest in the previous 3 frames (i.e., over 3 s of movement) and subsequent 3 frames. The error in this direction (i.e., the extent of deviation from 0, moving directly toward the nest) should decrease if looking forward is associated with making course corrections. Note that using different values (up to 30 degrees) for the definition of “forward looks” and different durations (1–5 frames) of averaging did not make a qualitative change to the reported results.

## 3. Results

**Figure 2:**
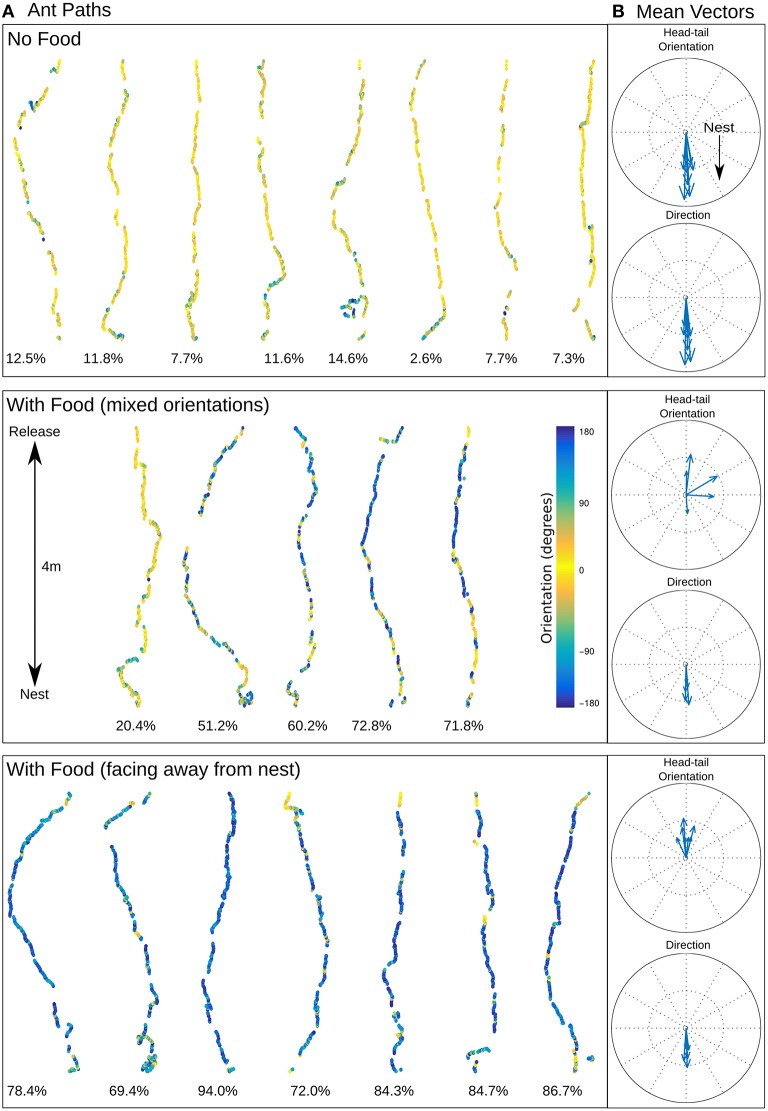
**Ant homing. (A)** Recorded ant paths, with head-tail orientation shown via color-coding for ants without food (top) and ants carrying prey (middle and bottom), and **(B)** mean vectors for each path for direction of movement and head-tail orientation. The mean direction for ants with or without food is almost identical (no food: mean 0.92 ± 56.40 degrees; with food (mixed orientations): mean 1.50 ± 68.94 degrees; with food (facing away from the nest): mean 1.80 ± 61.72 degrees) but the head-tail orientation is strikingly different (no food: mean 2.11 ± 47.32 degrees; with food (mixed orientations): mean 72.62 ± 131.88 degrees; with food (facing away from the nest): mean −177.59 ± 66.17 degrees). Below each path is shown the percentage of time the ant was moving backwards.

All 20 displaced ants returned directly to the nest irrespective of whether they were carrying food or not (Figure 2A). Although the ants had some path integration information, indicating the nest lay either north or south, there was no consistent deviation in their initial headings corresponding to this vector direction (Supplementary Material 1).

**Figure 3:**
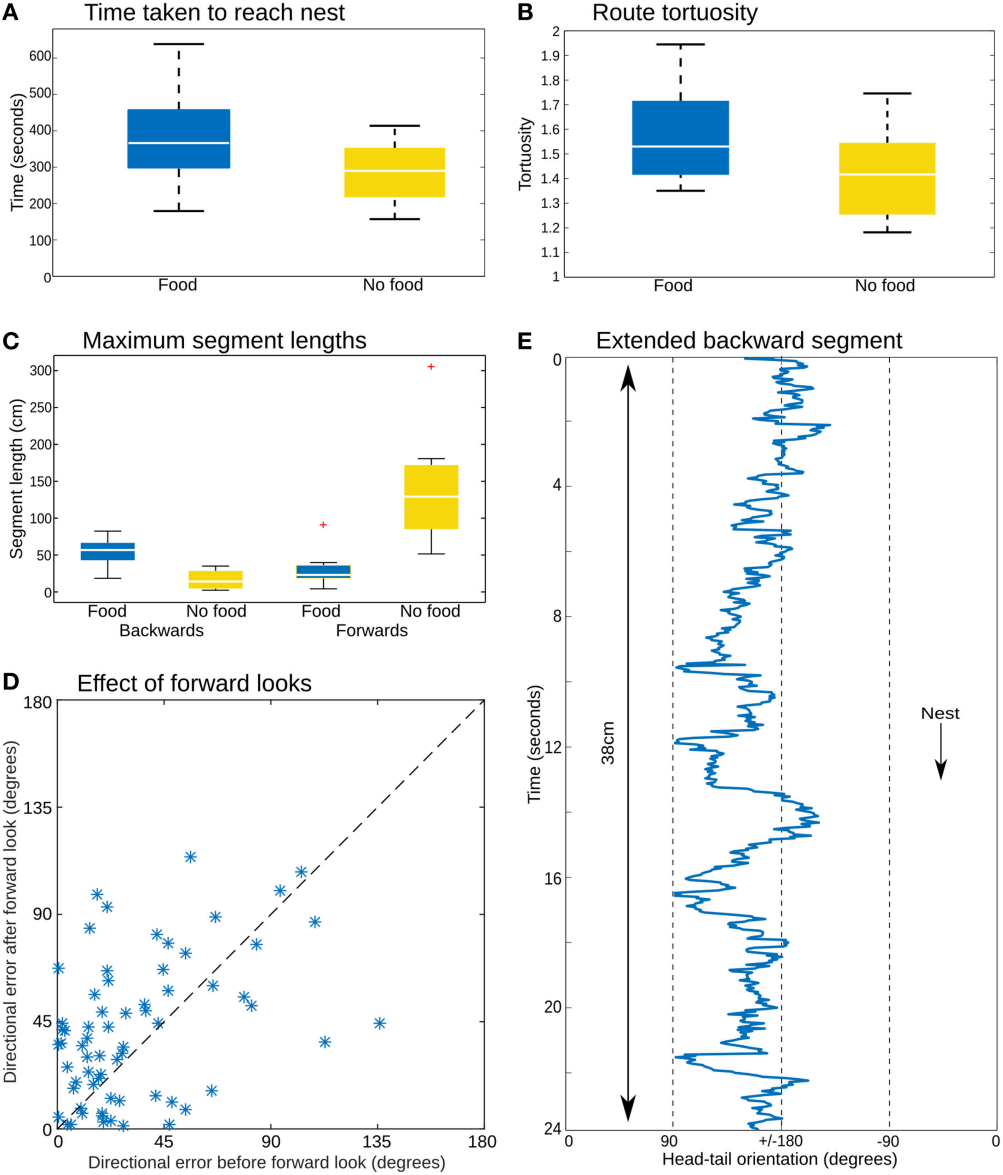
**Path analysis. (A)** The time to return to the nest is similar whether or not the animals were carrying a food item. **(B)** Path tortuosity was not significantly different. **(C)** While ants without food had distinctively longer segments facing toward the nest, the ants with food managed substantial distances without facing in the nest direction. **(D)** Comparing the direction of movement for three time steps before and after the moments at which prey-carrying ants face the nest (body-tail orientation <±5 degrees) shows no clear reduction in the error of the heading direction associated with such “forward looks,” which should appear as a preponderance of points below the *x* = *y* dashed line. **(E)** Head-tail orientation of a section of one ant path analyzed in every frame (25 Hz), showing an extended period of backward movement with no looks forward.

The color coding in Figure 2A shows the head-tail orientation relative to the nest of the ant at each point on the path. Ants without food generally faced toward the nest (yellow), but ants with large prey items still managed to travel toward the nest whilst spending significant amounts of time (percentage backward shown below each path) facing in completely the opposite direction (blue) due to the necessity to drag the food, including some long continuous segments without facing forward (Figures 3C,E). The ants carrying food thus had very similar mean direction of movement, despite extremely different head-tail orientation relative to the nest (Figure 2B). We found no statistical difference in the tortuosity of the paths of ants with and without food (no food: mean 1.42 ± 0.2, food: mean 1.58 ± 0.21, *t*-test 1.7214, *p* = 0.10, Figure 3B). In addition, the time taken to return to the nest is comparable for ants with and without food (control: mean 286.4 ± 87.5, experiment: mean 383.4 ± 123.6, *t*-test, 1.9144, *p* = 0.0716, Figure 3A). In each case the direction of difference is in favor of a faster and more direct return by ants not carrying food, and a larger N (in our experiment *N no food* = *8: N with food* = *12*) might have reached significance; but this could be equally due to the need to drag prey as to the higher percentage of backward movement. It remains evident that the food-dragging ants can maintain the home direction for significant distances without facing the nest. Thus continuous physical alignment toward the nest is not necessary to perform visual homing.

However, all ants show at least occasional moments of facing the nest, that is, none of them complete the full journey while facing only backwards. These nest-facing moments might provide ants with intermittent opportunities to acquire the bearing to the nest through retinotopic alignment. If this was coupled to some form of vector following or path stabilization mechanism, possibly with respect to celestial cues, it could explain the ant's ability to maintain their nestward direction when not aligned with the nest.

Previous reports have described stereotyped “stop and scan” behavior in ants (Wystrach et al., 2014; Zeil et al., 2014), particularly just after release in a novel environment. This was not obvious in our videos, where turning to face the nest occurred for a number of reasons, such as the need to manipulate the load over obstacles, or where the substrate was sufficiently smooth (such as across a leaf) to make a walk or jump forwards possible, and nestward facing periods appear to be equally distributed throughout the path (Supplementary Material 2). Nevertheless, some of these these moments of facing forward could be self-induced scans, or could at least provide opportunities to correct alignment through retinotopic matching or other methods. If this were the case, one might expect to see corrections in the course direction associated with these time points. However, comparison of the error in direction of ants, averaged over several seconds, before and after their forward-facing moments shows no evidence that the error has decreased after their “forward look” (Figure 3D). We note it is possible this analysis is not sufficiently sensitive to reveal corrections in the course. More detailed examination of the paths, or more direct experimental control over when ants can obtain view information may be needed to resolve this question.

## 4. Discussion

We have shown that visually guided ants can travel back to their nest after displacement to a novel location with little or no impairment of efficiency irrespective of the direction they are looking. This casts doubt on some current theories of how visual navigation is undertaken.

The use of continuous heading adjustment by retinotopic alignment, either through frequent scans to find the minimum in image difference (Baddeley et al., 2012) or oscillation around the minimum (Kodzhabashev and Mangan, 2015), has been successfully applied to mimic route following in ants. In this case, the ant is assumed to have stored images along previous traversals of the route, so is likely to have stored a nearby image, facing along the route, which will provide a clear minimum (or “look familiar”) when retinotopically aligned. Several papers (Graham et al., 2010; Narendra et al., 2013; Wystrach et al., 2013; Dewar et al., 2014; Zeil et al., 2014; Stürzl et al., 2015) describe how this approach could also explain visual homing behavior, if the ant is assumed to have performed learning walks to collect retinotopic views facing its nest from multiple directions around the nest. Our data suggests that this account is problematic, as ants can cover long distances toward the nest without facing the nest, and there is no apparent improvement in the directness of the approach for ants that mostly face the nest, or after the ant was “looking forward.”

A previous study of homing (Zeil et al., 2014), using the same ant species and a similar displacement approach to the current study, focussed on the initial movements of ants released in an unfamiliar location. These appeared consistent with the hypothesis that the ants use retinotopic alignment, at least to obtain an initial heading direction. It is thus possible that the ants in our study are using a “scan and fix” strategy. By taking an initial heading based on image matching, recording the compass direction of the nest using the celestial compass, and by flipping the bearing, the compass could be used to guide the path homeward while facing backwards. Or the ant could align to the nest, turn backward, record this new view, and use this to guide its direction. It may be the case that an even simpler mechanism is being used to maintain an approximately straight course between fixes, such as directional inertia (Lent et al., 2009) or optomotor stabilization (Götz, 1971).

However, we are not certain the ant could reliably obtain even its initial heading from the rIDF in this situation, at least if we assume the release location is novel (we cannot rule out that the ants in our experiment may have experienced the world from the location of the release platform while foraging). If the ant has stored images near the nest (during learning walks) while facing from multiple directions toward the nest, and is now rotating far from the nest, there will be multiple minima in the scan as it aligns in turn with each memory (Narendra et al., 2013). The difference between these minima could be small, and potentially swamped by any noise that might be introduced by lighting changes, tilt, head-angle induced by dragging a large object, etc. The difference is dependent on the ratio of the assumed nest-learning walk distance to the nest-release point distance, as illustrated in Narendra et al. (2013), Figure 7, where 1 m “learning walk” images from 4 cardinal directions produce almost identical minima for a 10 m scan whereas 5 m images produce one with a significantly deeper minimum. The available evidence on learning walks for these ants seems to us more consistent with the shorter distance (Jayatilaka, 2014). To date, successful use of retinotopic alignment for homing has been evaluated mostly in simulated or reconstructed environments, where noise is absent (Wystrach et al., 2013; Dewar et al., 2014; Zeil et al., 2014; Stürzl et al., 2015). The presence of this problem (i.e., lack of a single clear minima) for real images in Wystrach et al. (2012) led to the proposal that ants use different strategies when on a familiar route (rIDF retinotopic alignment) versus novel locations (skyline matching of absolutely aligned views), with the strategy selected through a visual familiarity threshold.

An alternative explanation for our results is that ants could be using the translational IDF to move toward the nest. As discussed in the introduction, this could potentially operate without physical alignment by using comparisons of the current image to a set of images stored at or around the goal in different orientations. As the ant adopts different headings, it is assumed that different images will provide a sufficiently good match such that the overall minimum should decrease as the goal is approached, and the ant could follow the gradient of this surface toward its nest. However, as the ant moves in and out of perfect alignment with one of its stored images, the surface becomes subject to local minima, surrounded by increases in the IDF when it is not so well aligned, as shown for example in Wystrach et al. (2012) where an absolute view alignment process was required for homing from novel location. Combined with other limitations of tIDF, such as the need to move through three non-colinear points to obtain sufficient gradient information to adopt an accurate direction, it is not clear that this method could account for our current data.

Following Baddeley et al. (2012), we have previously argued that simple retinotopic matching to all stored memories, through physical alignment to move in the direction that looks most familiar, can explain visual navigation in ants (Kodzhabashev and Mangan, 2015; Ardin et al., 2016). The current results suggest that a more complicated process is at work. For example, some of the described issues with the retinotopic alignment could be overcome by storing celestial compass directions to index retrieval of visual memory (Müller and Wehner, 2010). A further possibility is that the visual scene is stored in a form which is rotation and tilt invariant, which could allow IDF gradient descent despite the noise induced by uneven terrain and transport of food items (Ardin et al., 2015). At this stage it remains to be seen whether this hypothesis can explain our data, or whether an even more abstracted process, such as mental rotation, is at work in these amazing animals.

## Author contributions

PA, conducted the original research, performed the majority of the analysis, authorship of paper. MM, development of argument, authorship of paper. BW, supervised research, undertook analysis, authorship and review of paper.

## Funding

This work was supported in part by grants EP/F500385/1 and BB/F529254/1 for the University of Edinburgh School of Informatics Doctoral Training Center in Neuroinformatics and Computational Neuroscience (www.anc.ac.uk/dtc) from the UK Engineering and Physical Sciences Research Council (EPSRC), UK Biotechnology and Biological Sciences Research Council (BBSRC), and the UK Medical Research Council (MRC).

### Conflict of interest statement

The authors declare that the research was conducted in the absence of any commercial or financial relationships that could be construed as a potential conflict of interest.

## Supplementary material

Supplementary Material 1 **Release vectors**. The immediate paths taken by the animals on release was recorded while still on the platform. There is no obvious difference in the initial vector irrespective of whether the capture occurred North or South of the nest entrance. We take this as evidence that the effect of the path integration vector in our experiment was negligible.

Supplementary Material 2 **Path location and orientation**. The view direction was categorized as forwards (<±90 degrees of the nest) or backwards (>±90 degrees of the nest) and collated for each of the groups used in Figure 2A. The relative frequency of the two categories of view direction was calculated along the path using 10cm bins. The path length was 4 m but for both groups of ants with food this was occasionally exceeded when the animal circled around the nest entrance. While there is a greater number of forward looks at the start of the path for “backwards ants” the release platform has a smooth and level surface which in some cases allows the ant to walk forwards. Therefore we believe this data does not either support or refute the possibility of ants looking forwards and taking a fix on the nest direction at the start of their path.
