# Supplementary figures and images for: Ant Homing Ability Is Not Diminished When Traveling Backwards
Source: Front Behav Neurosci. 2016 Apr 13;10:69. doi: 10.3389/fnbeh.2016.00069 (PMC4829585; doi:10.3389/fnbeh.2016.00069)

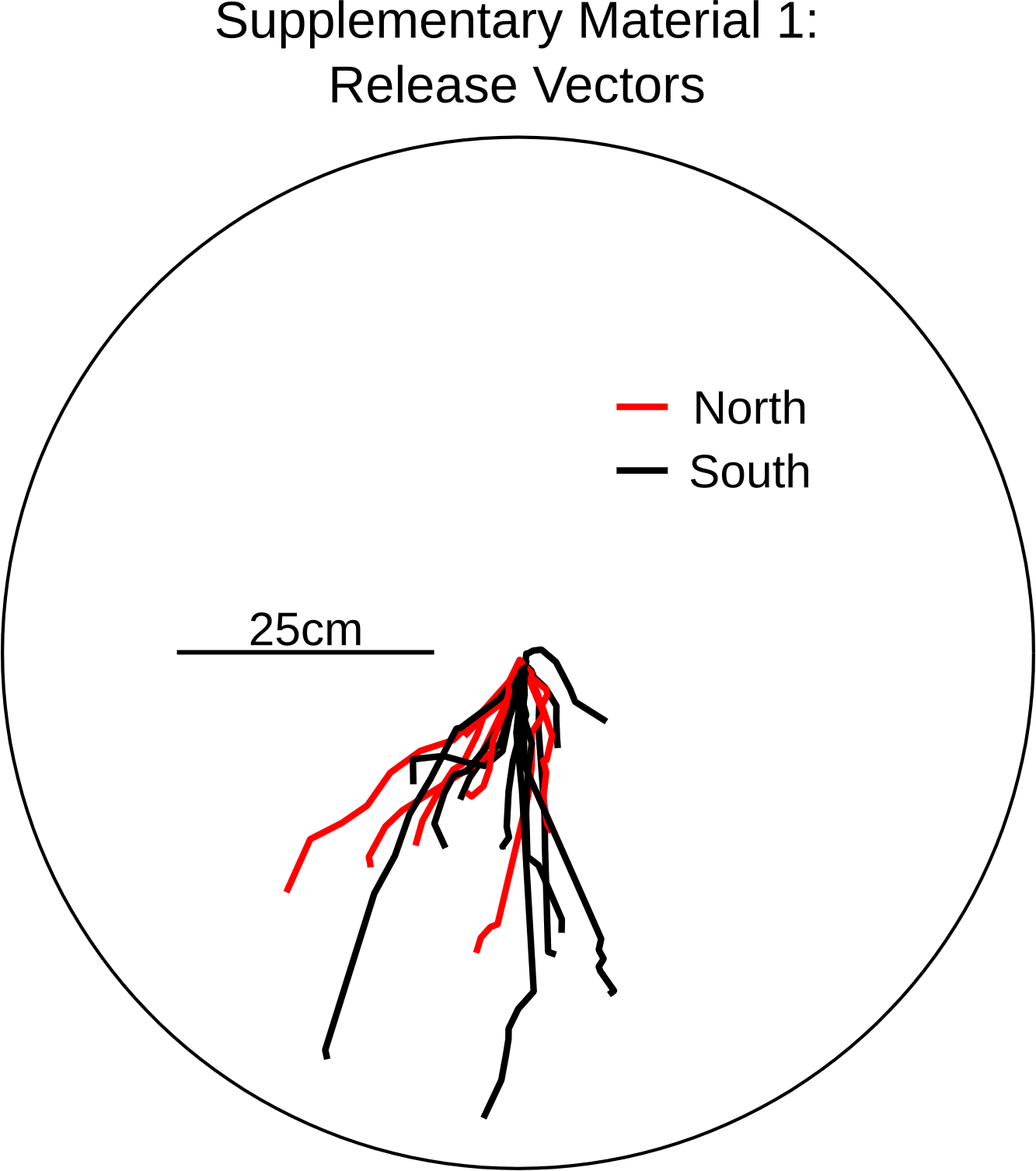

Supplement: Supplementary Material 1 — Release vectors. The immediate paths taken by the animals on release was recorded while still on the platform. There is no obvious difference in the initial vector irrespective of whether the capture occurred North or South of the nest entrance. We take this as evidence that the effect of the path integration vector in our experiment was negligible. [file Image1.TIFF]

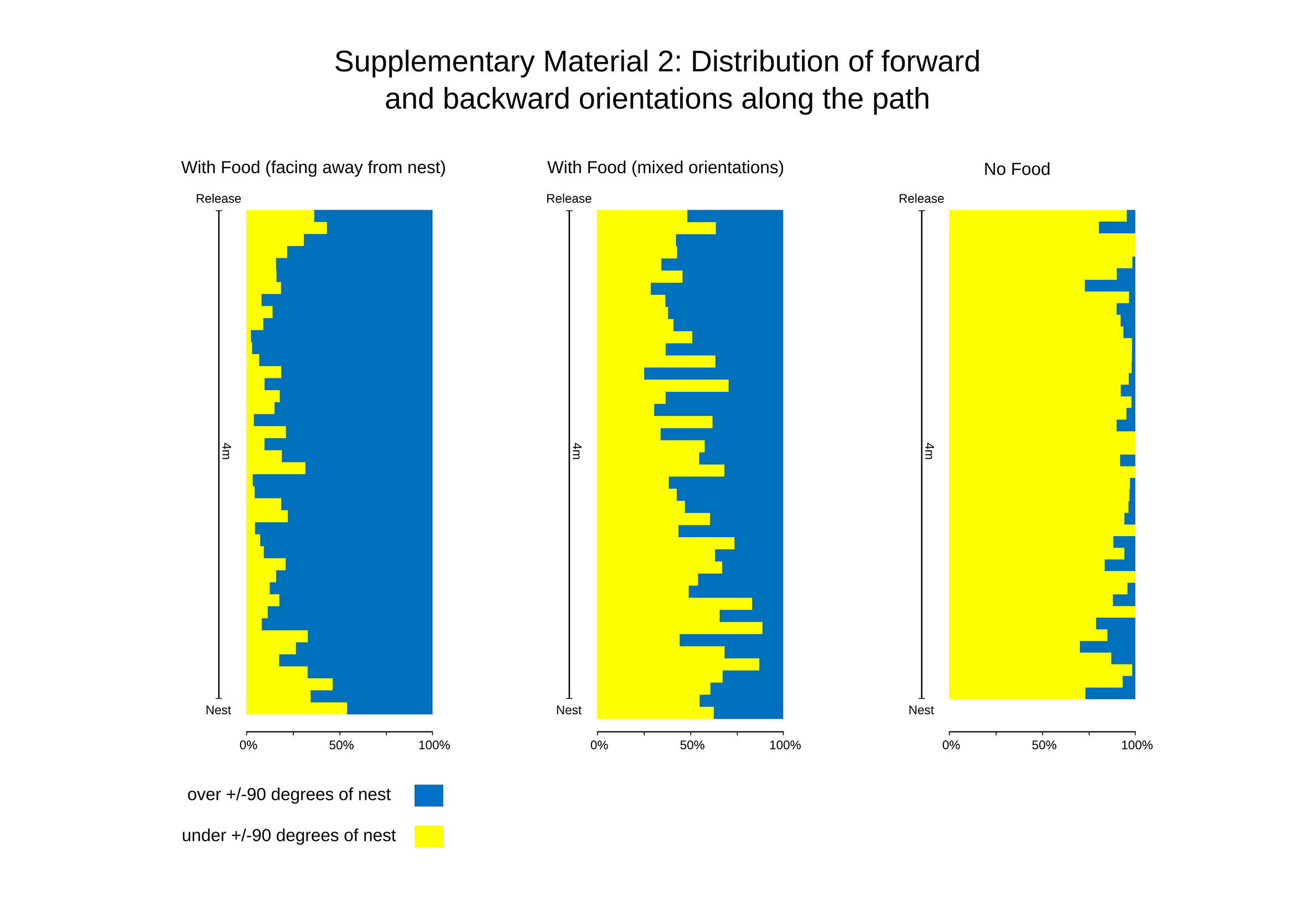

Supplement: Supplementary Material 2 — Path location and orientation. The view direction was categorized as forwards (<±90 degrees of the nest) or backwards (>±90 degrees of the nest) and collated for each of the groups used in Figure 2A. The relative frequency of the two categories of view direction was calculated along the path using 10cm bins. The path length was 4 m but for both groups of ants with food this was occasionally exceeded when the animal circled around the nest entrance. While there is a greater number of forward looks at the start of the path for “backwards ants” the release platform has a smooth and level surface which in some cases allows the ant to walk forwards. Therefore we believe this data does not either support or refute the possibility of ants looking forwards and taking a fix on the nest direction at the start of their path. [file Image2.TIFF]
